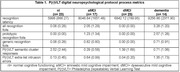# A Brief Digital Neuropsychological Protocol – II: Using Artificial Intelligence to Assess Verbal Serial List Learning Recognition Latency

**DOI:** 10.1002/alz.094303

**Published:** 2025-01-09

**Authors:** David J. Libon, Rodney Swenson, Sean E Tobyne, Catherine C. Price, Melissa Lamar, Stephanie Cosentino, Russell Banks, Ali Jannati, John Showalter, David Bates, Alvaro Pascual‐Leone

**Affiliations:** ^1^ Rowan University, Stratford, NJ USA; ^2^ University of North Dakota School of Medicine and Health Sciences, Grand Forks, ND USA; ^3^ Linus Health, Waltham, MA USA; ^4^ University of Florida, Gainesville, FL USA; ^5^ Department of Psychiatry and Behavioral Sciences, Rush University Medical Center, Chicago, IL USA; ^6^ The Taub Institute for Research on Alzheimer’s Disease and the Aging Brain, Columbia University, New York, NY USA; ^7^ Linus Health, Boston, MA USA; ^8^ Harvard Medical School, Boston, MA USA

## Abstract

**Background:**

Digital neuropsychological assessment easily captures behavior previously not obtainable by traditional pencil‐and‐paper tests. Verbal serial list learning tests are commonly used to assess for putative neurogenerative syndromes. Recognition test performance is often expressed compiling simple ‘yes/ no' responses, but fail to assess process metrics such as the latency to respond to individual recognition test items.

**Method:**

Memory clinic patients were assessed with the a digital neuropsychological protocol where cluster analysis of traditional metrics classified patients into normal (nl = 23), amnestic MCI (aMCI = 17), dysexecutive MCI (dMCI = 23), and dementia (dem = 14) groups. Verbal episodic memory was assessed with a 6‐word Philadelphia (repeatable) Verbal Learning Test. P(r)VLT delayed recognition memory was assessed using a forced multiple‐choice format where the iPad both displayed and verbally administered six trials containing the target word, a prototypic semantic foil (e.g., “apple”) and a generic semantic foil (e.g., “pear”). The patient was asked to touch the word that was part of the original word list as quickly as possible.

**Result:**

aMCI and dementia groups endorsed more recognition foils that other groups (Table 1; dementia > all groups, p< 0.001; aMCI > dMCI & nl groups, p< 0.001). Serial list learning recognition latency was slower for aMCI versus dMCI and nl groups; p< 0.005); and dementia versus dMCI and nl groups (p< 0.001). Regression analysis (dv = recognition latency; block 1 = age, education, sex; block 2 = recognition prototypic & generic foils) was significant (R2 = 0.463, p< 0.001); and found slower recognition latency was associated with greater numbers of prototypic recognition foils (beta = 0.551; p< 0.001). Regression analysis (dv = recognition latency; block 1 = age, education, sex; block 2 = all free recall cluster responses, all free recall extra‐list intrusion errors) also found slower recognition latency was associated with fewer semantic cluster responses (beta = ‐0.252, p< 0.021), but greater numbers of extra‐list intrusion errors (beta = 0.266, p< 0.017).

**Conclusion:**

When brought to scale, automated analysis of recognition latency along with other serial list learning process metrics could help identify early, emergent neurodegenerative illness.